# *SoxB2* in sea urchin development: implications in neurogenesis, ciliogenesis and skeletal patterning

**DOI:** 10.1186/s13227-018-0094-1

**Published:** 2018-02-19

**Authors:** Evgeniya Anishchenko, Maria Ina Arnone, Salvatore D’Aniello

**Affiliations:** Biology and Evolution of Marine Organisms, Stazione Zoologica Anton Dohrn Napoli, Villa Comunale, 80121 Naples, Italy

**Keywords:** Nervous system development, Ciliogenesis, Skeletal patterning, Evolution, Echinoderms

## Abstract

**Background:**

Current studies in evolutionary developmental biology are focused on the reconstruction of gene regulatory networks in target animal species. From decades, the scientific interest on genetic mechanisms orchestrating embryos development has been increasing in consequence to the fact that common features shared by evolutionarily distant phyla are being clarified. In 2011, a study across eumetazoan species showed for the first time the existence of a highly conserved non-coding element controlling the *SoxB2* gene, which is involved in the early specification of the nervous system. This discovery raised several questions about *SoxB2* function and regulation in deuterostomes from an evolutionary point of view.

**Results:**

Due to the relevant phylogenetic position within deuterostomes, the sea urchin *Strongylocentrotus purpuratus* represents an advantageous animal model in the field of evolutionary developmental biology. Herein, we show a comprehensive study of *SoxB2* functions in sea urchins, in particular its expression pattern in a wide range of developmental stages, and its co-localization with other neurogenic markers, as *SoxB1*, *SoxC* and *Elav*. Moreover, this work provides a detailed description of the phenotype of sea urchin *SoxB2* knocked-down embryos, confirming its key function in neurogenesis and revealing, for the first time, its additional roles in oral and aboral ectoderm cilia and skeletal rod morphology.

**Conclusions:**

We concluded that *SoxB2* in sea urchins has a neurogenic function; however, this gene could have multiple roles in sea urchin embryogenesis, expanding its expression in non-neurogenic cells. We showed that *SoxB2* is functionally conserved among deuterostomes and suggested that in *S. purpuratus* this gene acquired additional functions, being involved in ciliogenesis and skeletal patterning.

**Electronic supplementary material:**

The online version of this article (10.1186/s13227-018-0094-1) contains supplementary material, which is available to authorized users.

## Background

Many studies have been undertaken in order to unravel the developmental significance of *SoxB1* and *SoxB2* crosstalk in the animal kingdom [1–4]. These genes are structurally very similar, but have antagonistic roles: *SoxB1* is in fact considered a transcriptional activator, while *SoxB2* a transcriptional repressor [5]. In all bilateria, they are mainly involved in development and cell specification [6], and despite the fact that they participate to common processes, much attention has been paid to the role of *SoxB1* in development, while the knowledge of *SoxB2* functions is still quite limited. *SoxB2* is known to be a neurogenic transcription factor (TF), and its importance has been re-evaluated by the finding of an ultra-conserved *SoxB2* non-coding regulatory element discovered in distant metazoan phyla, from cnidarians to human [7].

The function of *Sox21*, the *SoxB2* vertebrate’s ortholog, has been mostly studied in fish, chicken and mouse. In chicken, it has a neurogenic function and it is expressed in vestibular and auditory organs, being an important regulator of sensory cell differentiation [8, 9]. In *Xenopus laevis*, *Sox21* is expressed in several regions of the central nervous system (CNS) and in the sensory organs [4], similarly to chicken [10]. Moreover, *Sox21* in zebrafish plays an important role in lens formation, embryonic CNS development, endoderm and ectoderm differentiation [11].

In vertebrates, it has been demonstrated the existence of functional redundancy among Sox family members [12, 13] and a major role in specification of several cell types and tissues seems to be due to their tendency to possess hypervariable *cis*-regulatory mechanisms. In invertebrate deuterostomes, *SoxB2* has been studied in acorn worm, *Ptychodera flava*, in which it is expressed in the ciliary band, apical organ and foregut [14]. In the cephalochordate *Branchiostoma belcheri*, *SoxB2b* is expressed in the neural plate and subsequently in the neural tube and foregut [15].

In the present study, we focused our attention on *SoxB2* during the development of the sea urchin *S. purpuratus*, belonging to echinoderms, which shares a common ancestor with modern chordates dating back about 500 million years [16]. Several studies were carried out in the purple sea urchin *S. purpuratus* at early developmental stages for maternally transmitted *SoxB2* [1, 17–19]. In particular, Kenny and colleagues showed *SoxB2* expression pattern and its putative function by knock-down experiments. They showed an involvement of *SoxB2* into the oral ectoderm formation and body axes establishment during sea urchins gastrulation [1]. A more recent study demonstrated the implication of *SoxB2* in neuronal specification up to 72 h post-fertilization (hpf) [19]. Nevertheless, there is still a lack of information regarding the *SoxB2* expression pattern at later developmental stages, as well as a detailed study regarding *SoxB2* non-neurogenic functions. Our study aimed to fill this gap in light of the recent comprehensive description of the sea urchin larval nervous system [20–24] showing for the first time the complete expression pattern including late developmental stages (144 hpf) and demonstrating its implication in multiple developmental processes, as NS specification, ciliogenesis and, intriguingly, skeletogenesis, the latter representing an echinoderm-specific ontogenetic mechanism.

## Results

### Nervous system specification during sea urchin development: orchestration by Sox genes expression

**Fig. 1:**
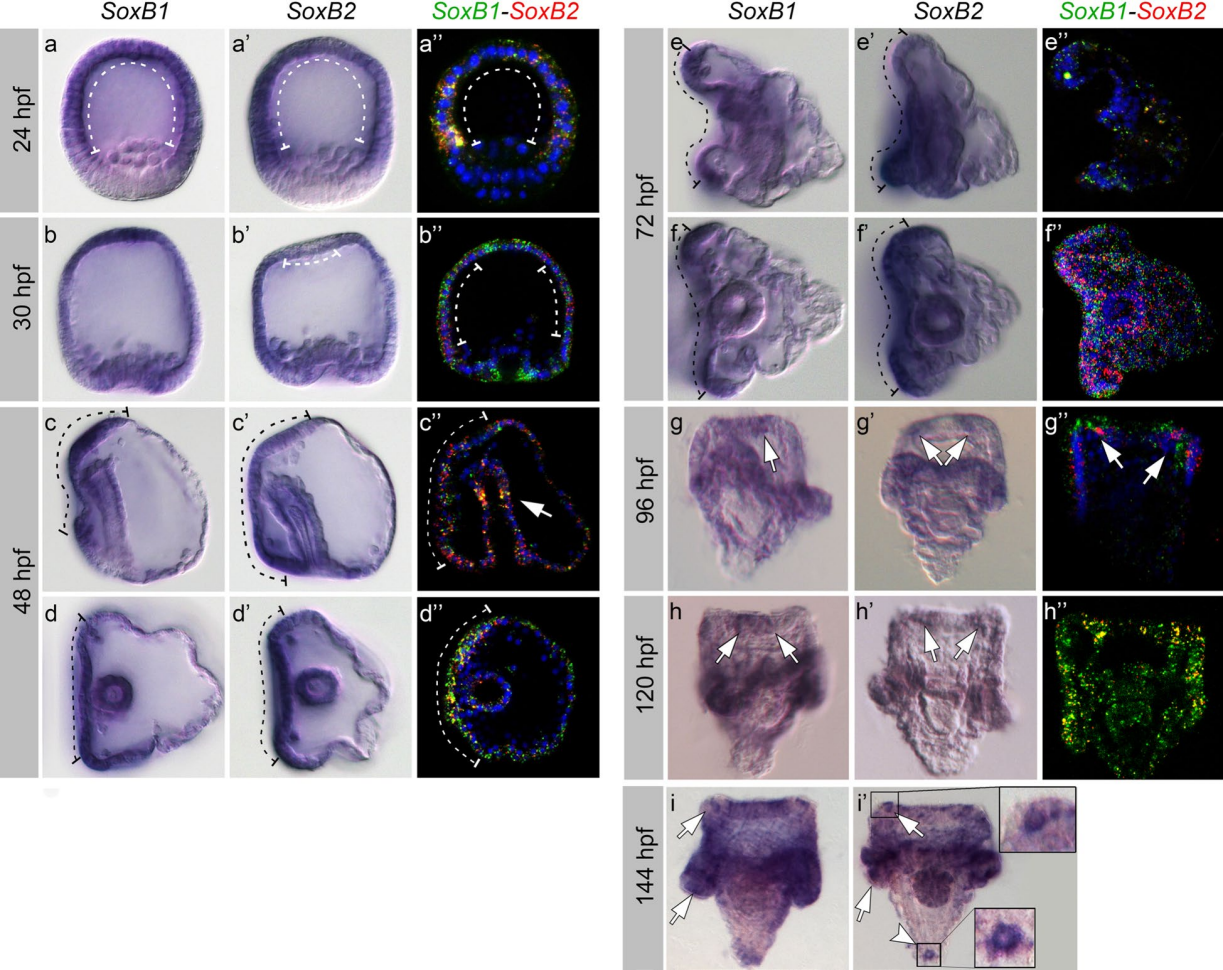
*SoxB1* and *SoxB2* genes expression in *S. purpuratus* during first week of development. Colorimetric (**a**–**a**′, **b**–**b**′, **c**–**c**′, **d**–**d**′, **e**–**e**′, **f**–**f**′, **g**–**g**′, **h**–**h**′, **i**–**i**′) and double fluorescent (**a**″, **b**″, **c**″, **d**″, **e**″, **f**″, **g**″, **h**″) *SoxB1* and *SoxB2* in situ hybridizations in embryos at 24–144 hpf. Fluorescent in situ hybridizations: *SoxB1* is green and *SoxB2* is red. The nuclear marker DAPI is shown in blue

While transcriptional data of *SoxB1* and *SoxB2* have been comprehensively included in the Echinoderms genome database (Echinobase) up to prism developmental stage, very little is known about late larval expression profile of these two genes. To fill this gap, we performed in situ hybridization experiments at blastula (24 hpf), early and late gastrula (30 and 48 hpf) and pluteus (72, 96, 120 and 144 hpf) stages, using both *SoxB1* and *SoxB2* riboprobes (Fig. 1). Moreover, although many studies addressed the interplay of these two genes in the developing nervous system [1, 3, 4], their co-localization was never shown before. By using double fluorescent whole mount in situ hybridization (WISH) of *SoxB1* and *SoxB2*, we analyzed their co-localization (Fig. 1a″–h″). At blastula stage, the expression patterns of *SoxB1* and *SoxB2* overlap in the ectoderm (Fig. 1a″, dashed line), with the exception of the vegetal pole. At early gastrula stage, *SoxB1* is ubiquitous but mostly expressed in the animal and vegetal ectoderm (Fig. 1b, b″). *SoxB2* has a similar expression pattern at this stage, except that it is absent in the apical ectoderm (Fig. 1b′, dashed line). *SoxB1* and *SoxB2* are co-expressed in the dorsoventral and left–right ectoderm (Fig. 1b″, dashed line). At late gastrula stage, both genes are expressed mainly in the oral ectoderm (Fig. 1c–c″, d–d″, dashed lines) and in the foregut (Fig. 1c″, arrow). At pluteus stage (72 hpf), both genes show strongest expression in the oral ectoderm around the ciliary band (Fig. 1e–e′, f–f′, dashed lines), and the aboral ectoderm lacks *SoxB1* and *SoxB2* expression. At 96 hpf, *SoxB1* and *SoxB2* are still expressed within the ciliary band, but *SoxB2* expression levels decrease in the apical organ (arrows in Fig. 1 g′–g″). Later in development (120 hpf), *SoxB1* partially disappears from tips of the oral arms (Fig. 1h, arrows) and *SoxB2* remain unchanged in this area (Fig. 1 h′, arrows). At 144 hpf, *SoxB1* and *SoxB2* expression in ciliary band can be detected only using colorimetric WISH (Fig. 1i–i′), thanks to prolonged exposure of the enzymatic reaction. At this late larval stage, the expression of both genes is prevalent in oral and aboral arms of the embryo as shown in Fig. 1i–i′ (arrows). Moreover, *SoxB2* appear strongly expressed in a single cell at the pluteus apex (Fig. 1i′, arrowhead).

**Fig. 2:**
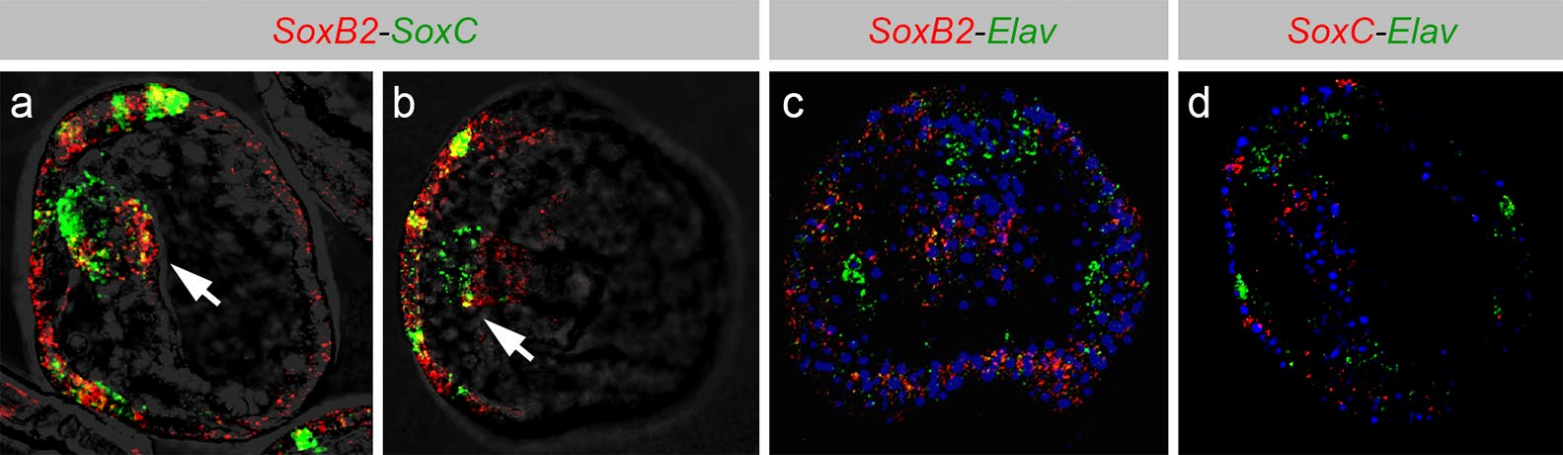
*SoxC, Elav* and *SoxB2* genes expression in *S. purpuratus* at 48 hpf. FISH in sea urchin embryos: *SoxC* is represented in green and *SoxB2* in red. Embryos are shown from lateral (**a**) and apical (**b**) views. Regions of co-expression are shown in yellow. **c** FISH in sea urchin embryos; *Elav* is green and *SoxB2* is red. Embryos are shown from the oral view. **d** FISH in sea urchin embryos; Elav is green and *SoxC* is red. Embryos are shown from lateral view. DAPI is shown in blue

Furthermore, to understand the crosstalk between *SoxB2* and other genes implicated in neuronal differentiation, we carried out double fluorescent in situ hybridizations (FISH) in order to assess the co-localization of *SoxB2* with neurogenic transcription factors, *SoxC* and *Elav*, which were previously described to have a sequential expression during sea urchins neurogenesis [25]. As Garner and colleagues recently showed by immunohistochemistry [19], we confirmed by FISH that some *SoxC* positive cells also express *SoxB2* in 48 hpf embryos in the oral ectoderm (Fig. 2a, b). We highlighted novel expression domain for both genes in adjacent cells within the foregut, which only partially co-localize (Fig. 2a, b, arrows). On the other hand, we tested the expression of *Elav* that is a terminal marker of neuronal specification and demonstrated that it does not co-express with *SoxB2* neither with *SoxC* (Fig. 2c, d).

### *SoxB2* functional and molecular characterization

**Fig. 3:**
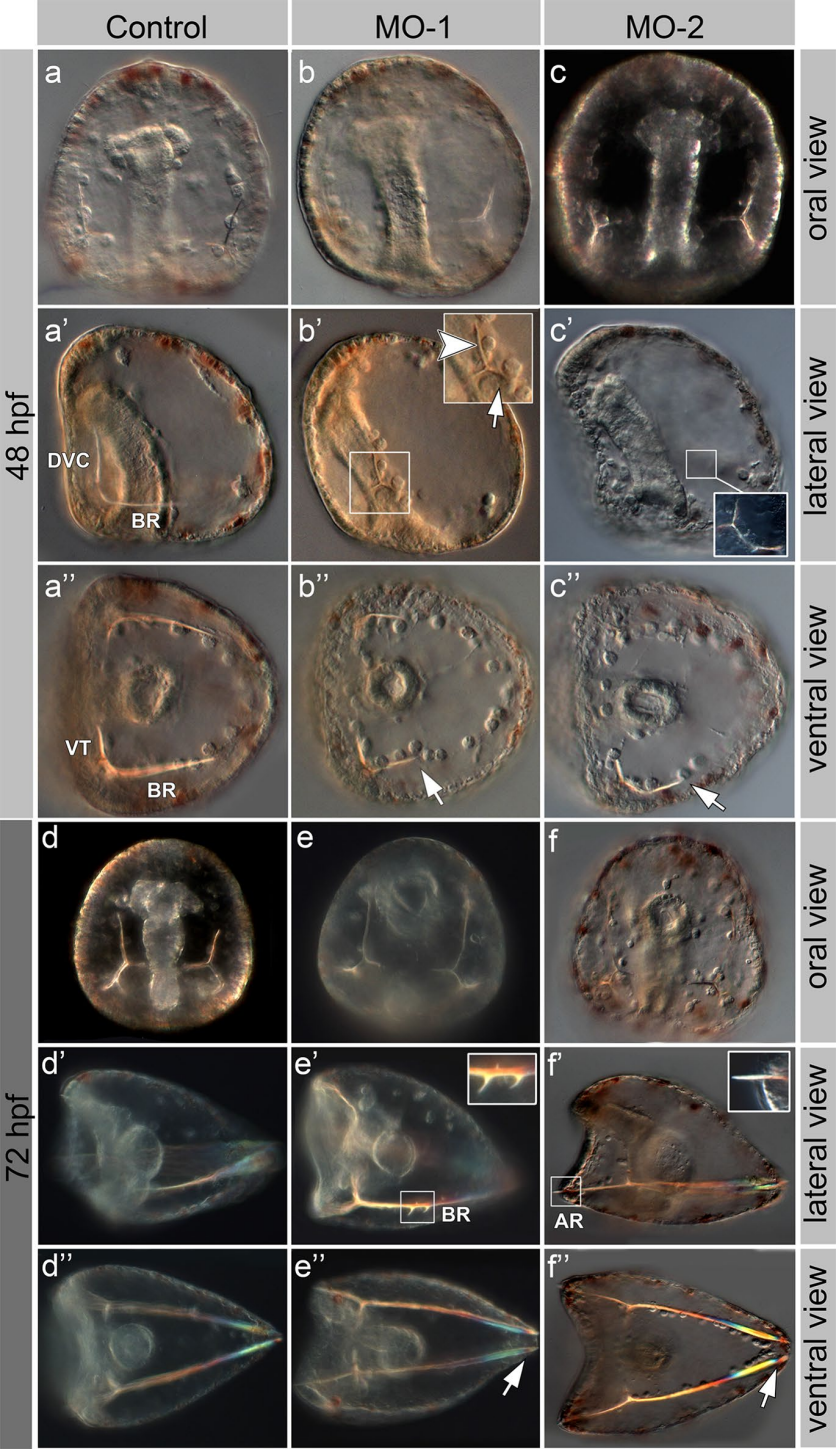
*SoxB2* MO-1 and MO-2 injection effect on skeleton formation in 48 and 72 hpf *S. purpuratus* embryos. Oral (**a**, **b**, **c**, **d**, **e**, **f**), lateral (**a**′, **b**′, **c**′, **d**′, **e**′, **f**′) and vegetal (**a**″, **b**″, **c**″, **d**″, **e**″, **f**″) view of embryos at 48 hpf (**a**–**c**″) and 72 hpf (**d**–**f**″). Uninjected control embryos (**a**–**a**″ and **d**–**d**″) were compared with morphants, injected with *SoxB2* MO-1 (**b**–**b**″, **e**–**e**″) and *SoxB2* MO-2 (**c**–**c**″, **f**–**f**″)

Previous studies showed that *SoxB2* knock-down led to body axes alterations and gastrulation arrest [1]. Herein, we used identical morpholino antisense oligonucleotides (MO) sequences that, injected at a lower concentration compared to what reported in [1], led to a less severe phenotype, allowing gastrulation to occur and revealing other developmental defects. The analysis of the obtained phenotype permitted to describe additional developmental processes that require *SoxB2* activity. We monitored the general morphology and nervous system development of *SoxB2* morphant embryos during the first 72 hpf. The most evident phenotype in 48 and 72 hpf *SoxB2* morphants was a prevalence of embryos with roundish-shaped body showing defects in the characteristic oral ectoderm (Fig. 3b–c″, e–f″). Noteworthy, at 48 hpf *SoxB2*-MOs affect dorsoventral connecting rods (DVC) and body rods (BR) which are shorter than in controls (Fig. 3b′,b″; arrowhead and arrows, respectively). Body rods appear slightly curved (Fig. 3c″, arrow), while ventral transverse rods (VT) in MO-1 and MO-2 embryos remain similar to those in controls (Fig. 3a″–c″, arrows). Later in development, at 72 hpf, several defects have been observed such as additional ramification of BRs (Fig. 3e′), outgrowth of posterior anal rods (AR) (Fig. 3f′) and abnormal growth of the BRs that do not converge in the apex as it normally occurs (Fig. 3e″, f″, arrows) [26, 27].

We injected two different *SoxB2* morpholino antisense oligonucleotides: MO-1 (identical to the one used by Kenny and colleagues [1] and MO-2, which evoked similar phenotypes in at least 97% of injected embryos (Fig. 3b–b″, e–e″, c–c″, f–f″, respectively). The efficiency of our microinjection experiments and the specificity of MO effects were controlled using a fluorescein-tagged standard control MO (MO-Fluo) that showed green fluorescent signal in 95% injected embryos. The morphology of sea urchin embryos injected with MO-Fluo was comparable to the uninjected controls, and no skeletal defects were ever observed in such control injection performed side by side with *SoxB2* MO-1 and MO-2 injection on the same batch of embryos (Additional file 1: Figure S1). The survival rate of all injected embryos was > 90%.

**Fig. 4:**
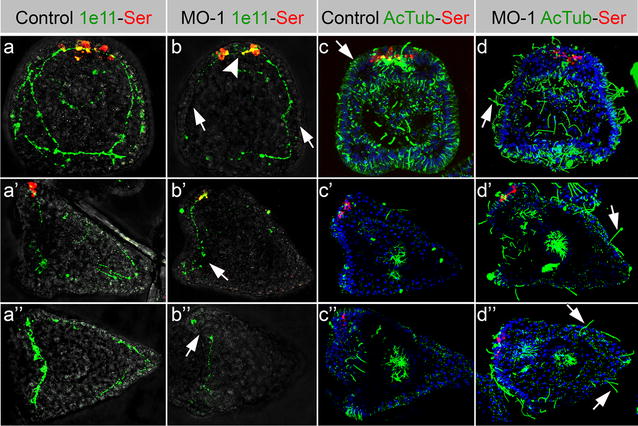
*SoxB2* MO-1 injection effect on nervous system formation and ciliogenesis in 72 hpf *S. purpuratus* embryos. Oral (**a**, **b**, **c**, **d**), lateral (**a**′, **b**′, **c**′, **d**′) and vegetal (**a**″, **b**″, **c**″, **d**″) views of embryos at 72 hpf. Uninjected control embryos (**a**–**a**″ and **c**–**c**″) were compared with morphants, injected with *SoxB2* MO-1 (**b**–**b**″, **d**–**d**″). Serotonin is shown in red, DAPI in blue, Synaptotagmin B (1e11) in **a**–**b**″ and AcTubulin in **c**–**d**″ are shown in green

The detailed analysis of NS and ciliary band formation in *SoxB2* morphants was performed in embryos at 72 hpf by immunohistochemical analysis (IHC) using anti-acetylated tubulin (AcTub) to stain ciliated structures, anti-serotonin (Ser) and anti-synaptotagmin B (1e11) to detect neuronal networks. NS alterations were observed in *SoxB2* morphants, in fact 72 hpf sea urchin embryos injected with *SoxB2* MO-1 showed that the number of synaptotagmin B positive cells was significantly decreased (> 50%), mostly at the expense of ganglial cells (Fig. 4b–b″, arrows; and Additional file 2: Figure S2a) compared to uninjected controls (Fig. 4a–a″) and to MO-Fluo injected embryos (Additional file 3: Figure S4d). As shown in Fig. 4b, the nerve ring, associated with the ciliary band and normally encircling the border of the oral ectoderm, appeared incomplete in pluteus stage (72 hpf) morphants, when compared with controls (Fig. 4a). Moreover, many neuronal cells lost axonal connections. The neuronal circle around the mouth was altered in morphants (Fig. 4b, arrows), and neurites did not project toward the posterior end of the larval body, compared to the control embryos (Fig. 4a′,b′). The number of serotonergic neurons decreased of 40% in the sea urchin *SoxB2* morphant larvae (Fig. 4b, arrowhead; Additional file 2: Figure S2b). This phenotype was detected in 85% of the injected embryos in six biological replicates.

Furthermore, our analysis showed that the morphants presented significantly longer cilia (21 ± 2 µm), compared to uninjected controls (11 ± 1 µm) (Fig. 4c–d″; Additional file 2: Figure S2c). Interestingly, longer cilia were observed even outside the innervated ciliary band over all the embryos body (Fig. 4d′–d″, arrows). The observed differences in cilia length are shown in Additional file 2: Figure S2c and Additional file 4: Figure S3. Control embryos, injected with 200 µM MO-Fluo, did not show significantly longer cilia compared to uninjected controls, confirming the specificity of *SoxB2* knock-down phenotype (Additional file 4: Figure S3d).

In order to enrich our knowledge on *SoxB2* function in sea urchin embryogenesis and to propose its possible molecular mechanisms, we integrated our study including expression levels of genes fundamental for ectoderm, nervous system, ciliary band formation, and skeletogenesis. To this aim, we performed qPCR analysis on mRNA extracted from 48 and 72 hpf *SoxB2* morphant embryos, compared with uninjected controls, to study the transcriptional alteration of selected ectodermal genes: *Onecut*, *SoxB1*, *SoxC, Pax2/5/8;* mesodermal genes*: Vegf, Bmp3, SM30, SM50*; and an ecto-endodermal gene: *Brn1/2/4.*

**Fig. 5:**
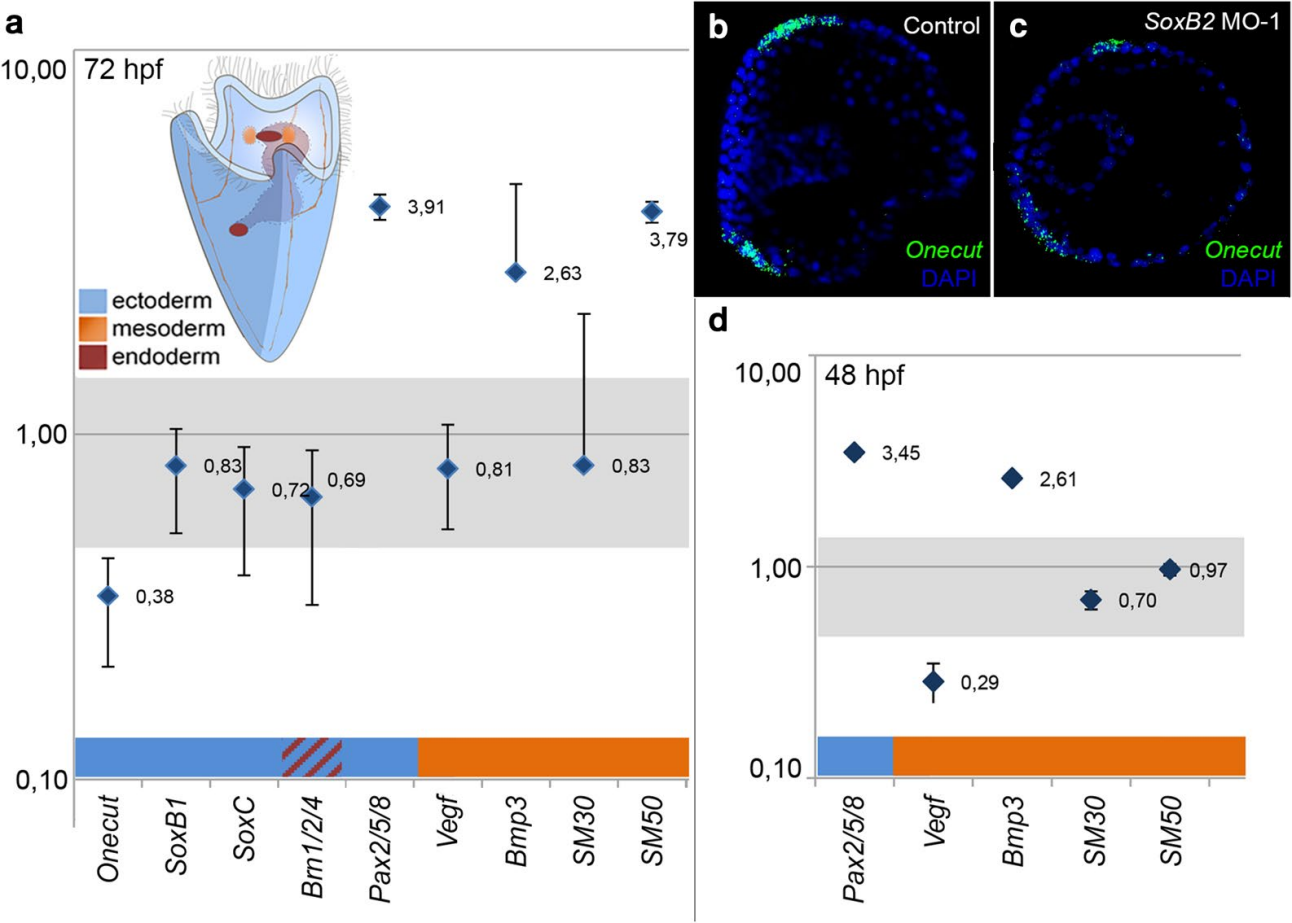
Expression levels of developmental genes in *S. purpuratus SoxB2* morphants. Ratio of gene expression levels comparing *SoxB2* morphants and uninjected control embryos at 72 hpf (**a**) and 48 hpf (**d**) by qPCR. Significant alterations in gene expression are below and above the gray bar (value ± 0.5). The histograms are represented in a Log10 scale for the y-axis. Decreasing levels of *SoxB1* (green) and *Onecut* (red) expression in *SoxB2* morphants was shown using FISH (**c**). Morphants were compared with uninjected control sea urchin embryos at 48 hpf, the images were taken from the apical view (**b**)

Among all neurogenic genes taking part in ciliary band formation *Onecut* is the only one that appeared significantly decreased in *SoxB2* morphants according to qPCR (Fig. 5a) and WISH experiments (Fig. 5b, c). *Vegf,* a gene known for its key role in sea urchin larval skeleton formation [26] resulted downregulated, while *SM50*, *Pax2/5/8* and Bmp3 showed a strong upregulation (Fig. 5a, d).

## Discussion

In the present work, we demonstrated that in sea urchin *S. purpuratus* embryos *SoxB2* and its paralog *SoxB1* co-localize at different developmental stages. We also showed their expression patterns at late developmental stages (96–144 hpf) and *SoxB2* co-localization with *SoxC* and *Elav*. *SoxB2* and *SoxB1* temporal and spatial expression patterns are quite similar at blastula and gastrula stages, and they co-occurred in the ectoderm at blastula stage and additionally appeared in foregut cells at gastrula stage. These data confirm previously known expression patterns of *SoxB1* and *SoxB2* [1, 2], even though the co-expression of these two genes had not been shown before. The main differences in their embryonic localization become clear at 30 hpf, when the vegetal pole and the animal pole domains (APD) [25, 27] express *SoxB1*, while APD lacks *SoxB2,* in agreement with what already shown by Garner et al. [19] at 48 hpf. At this developmental stage, *SoxB2* appears expressed (and co-localized with *SoxB1*) in oral ectoderm and in foregut, which has endodermal origin. Therefore, the expression of *SoxB2* in sea urchins is not limited to ectodermal tissues. This appears as an evolutionarily conserved feature, since endodermal expression of this gene has been already observed in chordates (amphioxus) [15]. Sea urchin foregut neurons are known to differentiate *in loco*; therefore, they do not derive from ectodermal precursors neither from migrating APD cells [18]. We here speculate that *SoxB2* could be a potential regulator of neurogenesis in the foregut. This hypothesis is reinforced by double WISH experiments demonstrating that *SoxB2* and the late nervous system marker, *SoxC*, are expressed in different cells within the foregut (Fig. 2a, b, arrow), reflecting an intermediate state of progression of foregut neurons maturation, with *SoxB2* playing a role in its initial phase. APD cells, in fact, express *Elav* that is a terminal factor of neuronal specification [19], and do not express *SoxB2* or *SoxC*. Hence, these data could have an impact in our understanding of the timing and hierarchy of foregut innervation.

The key role of *SoxB2* in neurogenesis was confirmed by the MO knock-down at 72 hpf that showed developmental abnormalities in oral neuronal ring with incomplete axonal connections and in ciliated structures that resulted longer than control embryos and, unlike controls, distributed along the whole body. These alterations were confirmed by qPCR analysis in which the ciliary band specification gene *Onecut* appeared as the only gene altered among proneural ectodermal genes tested in our experiments, while *SoxB1, SoxC* and *Brn1/2/4* remained unaffected. WISH experiments showed that *Onecut* expression domain was significantly reduced (Fig. 5).

In addition, we characterized the *SoxB2* expression pattern at late developmental stages, 144 hpf pluteus stage, in which the NS is already formed and SoxB2 acquires a new expression domain in the apex, where important morphogenetic processes shaping the skeleton occur at late pluteus stage. Therefore, *SoxB2* expression profile in *S. purpuratus* embryos suggests an alternative function to neurogenesis, suggesting implication in endoskeleton patterning. This hypothesis is further supported by the *SoxB2* knock-down phenotype. In fact, *SoxB2* morphants showed the reduction of spicules at 48 hpf and later the ramification and extension of the ventral spicules over the body at 72 hpf accompanied by alterations in their orientation and failure to convergence at the apex. The observed overexpression of *Vegf*, *Bmp3* and *SM50* (sea urchin embryo spicules) in *SoxB2* morphants positively correlates with the aberration of the endoskeleton structure (Fig. 5). In fact, *Pax2/5/8*, known to control primary mesenchyme cells specification and skeletogenesis via *Vegf, Bmp3, SM30* and *SM50* [28], resulted overexpressed in *SoxB2* morphants.

Many efforts have been dedicated in sea urchin developmental biology to understand individually the formation and regulation of skeletal patterning [28] as well as oral ectoderm specification [20, 25, 29] and differentiation of the innervated ciliated band [19, 24]. Based on experimental evidences reported in the present work, we hypothesized that *SoxB2* could be the cross-linking GRN factor that orchestrates the developmental and functional organization of derivatives from all three embryonic layers in sea urchin embryos: nervous system (ectoderm), foregut (endoderm) and endoskeleton (mesoderm). Nevertheless, in other animals, SoxB2 is prevalently a key regulator of the nervous system specification, as shown in cnidarian *Nematostella vectensis* [30, 31], in fruit fly *Drosophila melanogaster* [7], in hemichordate *Ptychodera flava* [14] and in chordates amphioxus [15, 32] and vertebrates [5, 6, 9, 10]. The only other example of non-neuronal roles so far described in other metazoans is represented by the amphioxus SoxB2 that has been shown to be involved in the embryonic gut [15, 32]. Therefore, it can be speculated that the *SoxB2* gene has been co-opted in sea urchin skeletogenesis.

## Conclusions

*SoxB2* in metazoans has a broad function in orchestrating NS at early stages of development. We here show that, at certain developmental stages, *SoxB2* in sea urchin embryos acquires secondary regulatory roles in foregut innervation, skeletogenesis and ciliogenesis. Moreover, the example of *SoxB2* role in sea urchin skeletogenesis bring to the discussion the Evo-Devo concept that novelty in evolution can also rise from ultra-conserved and stereotyped gene pathways without drastic genomic rearrangements. Interestingly, within echinoderms, the embryonic skeletogenesis is a lineage-specific process exclusively observed in Echinoids and Ophiuroids [33, 34]. Therefore, it would be interesting, in the future, to further investigate the putative co-option event that allowed *SoxB2* to acquire exclusive features in echinoderms.

## Methods

### Animal husbandry and embryo culture

Adult *S. purpuratus* were provided by Patrick Leahy (Kerchoff Marine Laboratory, California Institute of Technology, Pasadena, CA, USA) and housed in circulating seawater aquaria at 16 °C at the Stazione Zoologica Anton Dohrn, Napoli (Italy). Gamete release was induced by vigorously shaking ripe animals. The embryos were cultured in 0.22 µm filtered Mediterranean sea water, diluted with deionized H_2_O in a 9:1 ratio.

### Whole mount in situ hybridization

*SoxB1* (AF157389; SPU_022820), *SoxB2* (ABI53357.1; SPU_025113) and *SoxC* (NP_001158501; SPU_002603) cDNA were obtained by RT-PCR on total RNA that was isolated from 72 hpf *S. purpuratus* embryos using RNAqueous-Micro Kit (Ambion). The retro-transcription was carried out with the VILO SuperScript cDNA Synthesis kit (Invitrogen). cDNA amplification was performed using following primers: *SoxB1*-F: 5′-GTC TGT TCC TGG TGT ACA TG-3′; *SoxB1*-R: 5′-TAC ATA TGC GTG AGT GGA AC-3′; *SoxB2*-F: 5′-ATG ATG ATG GAC TCT GCG ATG G-3′; *SoxB2*-R: 5′-AGA GAG CCG TCG CCG CTG TCG G-3′; *SoxC*-F: 5′-GTT CCT CAG AAG AGC TTC GC-3′; *SoxC*-R: 5′-AGC AAT CGT CCA TGT CGA C-3′. cDNA fragments were cloned into p-GEM-T Easy vector (Promega). *Onecut* riboprobe was prepared using the sequence published by Poustka et al. [29]. *Elav* riboprobe was synthesized using a clonal plasmid provided by Dr. Paola Oliveri (University College London, UK; unpublished data). Gene clones were used for RNA probe synthesis with DNaseI-RNAse (Roche) and labeled with Digoxigenin or Fluorescein (Roche), according to the supplier’s protocol. For single colorimetric in situ hybridizations, we followed the protocol as was previously described [35] with following modifications: hybridization with RNA probe was performed at 63 °C overnight in 50% Formamide hybridization buffer. Double fluorescent in situ hybridization was performed as described in Cole et al. [36]. For differential interference contrast images, a Zeiss Axio Imager M1 microscope equipped with an “Axiocam” digital camera was used. Fluorescence images were obtained using a Zeiss confocal laser scanning LSM 510 microscope.

### Immunohistochemistry

72 hpf embryos were fixed and used for immunohistochemistry procedure as outlined in Burke et al. [21]. In order to localize acetylated tubulin, serotonergic and synaptotagmin B positive cells we used 1:400 diluted mouse monoclonal anti-acetylated tubulin antibody (AcTub; T7451, Sigma-Aldrich), rabbit monoclonal anti-serotonin antibody (Ser; S55451E11, Sigma-Aldrich) and 1:50 diluted mouse monoclonal anti-synaptotagmin B antibody (1e11) [21], respectively. The fluorescent staining was developed using goat anti-Mouse IgG Alexa Fluor^®^ 488 conjugate (A-110010, Invitrogen) and Donkey anti-Rabbit Alexa Fluor^®^ 555 conjugate (A-31572, Invitrogen) (1:500) secondary antibodies. Lastly, the embryos were incubated with nuclear marker DAPI (Sigma-Aldrich) 1:10000 in PBT (phosphate-buffered saline; 0.1% Tween 20).

### Knock-down by morpholino antisense oligonucleotides microinjections

Morpholino antisense oligonucleotides against *SoxB2* translation were designed and acquired from Gene Tools: MO-1: 5′-TCC CCA TCG CAG AGT CCA TCA TCA T-3′; MO-2 5′-GTC GGA TGC TGG CTT TCA AAA CAG A-3′). MOs were injected in approximately 500 embryos in quantity of 2 pl in each embryo. Injected solution contained 200 μM MO and 0.12 M KCl. Fluorescein-tagged standard control MO (MO-Fluo: 5′-CCT CTT ACC TCA GTT ACA ATT TAT A-3′) was used as a control and injected at 200 μM concentration in 250 embryos in all experiments. Embryos injected with MO-Fluo showed bright fluorescence; their phenotype corresponds to the phenotype of uninjected embryos at 20–96 hpf. Experiments were repeated at least six times. The survival rate of 200 μM MO-1 and MO-2 injected embryos was > 90% in all experiments. The morphant phenotype was observed in > 97% of injected survived embryos.

### Quantitative PCR

The qPCR was carried out in a ViiATM 7 Real-Time PCR System (Applied Biosystems) using the SYBR™ Green reagent (Life Technologies). The expression levels of several sea urchin developmental genes that could be affected by *SoxB2* (*Onecut*, *SoxB1*, *SoxC, Brn1/2/4*, *Pax2/5/8*, *Vegf, Bmp3, SM30* and *SM50*) were explored by qPCR using cDNA derived from 300 morphant embryos at 48 and 72 hpf injected with 200 μM *SoxB2* MO-1. cDNA was synthesized using RNAqueous-Micro Kit (Ambion). *Ubiquitin* and *Ef1a* were used for data normalization [37, 38]. Primer sequences used in the qPCR analyses are listed in Additional file 5: Table S1. Gene expression levels of the morphants were compared with those of uninjected embryos. Each qPCR experiment was performed on three independent biological replicas, and each reaction was repeated three times.

## Additional files

**Additional file 1: Figure S1.** Control MO-Fluo in 72 hpf *S. purpuratus* embryos. Sea urchin embryos at 72 hpf from oral (**a**, **b**, **c**), lateral (**a**′, **b**′, **c**′) and vegetal (**a**″, **b**″, **c**″) views depict the tissues where fluorescence deriving from the fluorescent MO is visible (**c**–**c**″). Morphant embryos imaged with microscope (**b**–**b**″) present a phenotype similar to uninjected control embryos (**a**–**a**″).

**Additional file 2: Figure S2.** Statistical analysis of serotonergic neurons number and cilia length in 72 hpf *S*. *purpuratus* *SoxB2* knock-down experiments. **a** Embryonic nervous system (Synaptotagmin B by 1e11 immunohistochemistry) of uninjected control, MO-1, MO-2 and MO-Fluo embryos. The fluorescence of 1e11 positive neurons is shown in %, normalized by control uninjected embryos (100%). The intensity of staining from 10 embryos of each group was measured using ImageJ in three independent experiments. **b** Number of serotonergic neurons observed in six independent experiments using uninjected control, MO-1, MO-2 and MO-Fluo embryos. Serotonergic positive neurons were measured from at least 33 embryos in each experimental group. **c** Cilia length in uninjected control, MO-1, MO-2 and MO-Fluo embryos measured using the Zeiss confocal laser scanning LSM 510 microscope software. 10–12 cilia from at least 33 embryos were used in three independent experiments. Statistical analysis was performed using Prism 5 GraphPad software: *P* value versus uninjected controls = *P < 0.05, **P < 0.01, ***P < 0.001, while *P* value versus MO-Fluo = ^+^*P*<0.05, ^++^*P* < 0.01, ^+++^*P* < 0.001.

**Additional file 3: Figure S4.** Injection of MO-Fluo did not affect the development of Synaptotagmin B expressing neurons.

**Additional file 4: Figure S3.** Analysis of the cilia length in MO-injected embryos performed at 72 hpf pluteus. AcTubulin staining is shown in green and DAPI in blue. **a** control non injected embryo, **b** MO-1 injected embryo, **c** MO-2 injected embryo, **d** MO-Fluo injected embryo. Measurements of longest cilia length (l) are indicated in white. All cilia length measurements were performed using Ziess LSM Image Browser software. 10–12 cilia from at least 33 embryos in every experimental group were measured; scale bar is 20 µm.

**Additional file 5: Table S1.** List of oligonucleotides used for qPCR experiments.

## Abbreviations

AcTub: acetylated tubulin
bp: base pairs
DAPI: 4′,6-diamidino-2-phenylindole
CNS: central nervous system
DIG: digoxigenin
FISH: fluorescent in situ hybridization
MO: morpholino antisense oligonucleotide
MO-Fluo: fluorescein-tagged standard control MO
hpf: hours post-fertilization
IHC: immunohistochemistry
NS: nervous system
qPCR: quantitative polymerase chain reaction
Ser: serotonin
TF: transcription factor
WISH: whole mount in situ hybridization
