# Supplementary figures and images for: SoxB2 in sea urchin development: implications in neurogenesis, ciliogenesis and skeletal patterning
Source: EvoDevo. 2018 Feb 19;9:5. doi: 10.1186/s13227-018-0094-1 (PMC5817722; doi:10.1186/s13227-018-0094-1)

Control 72 hpf

MO-Fluo 72 hpf

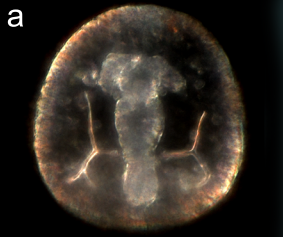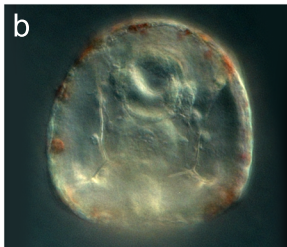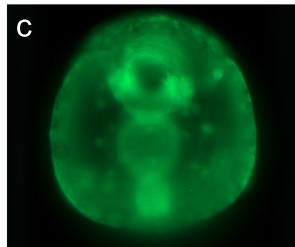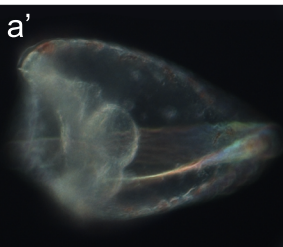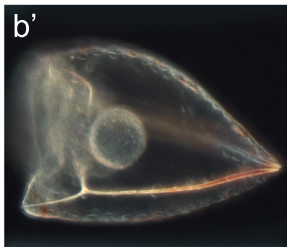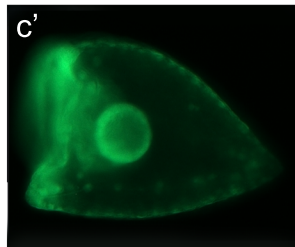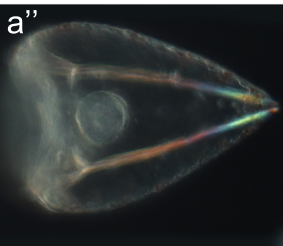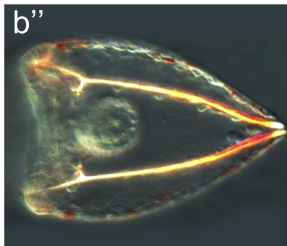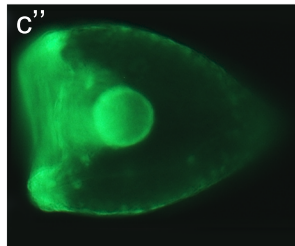

Supplement: Supplementary file 1 — Additional file 1: Figure S1. Control MO-Fluo in 72 hpf S. purpuratus embryos. Sea urchin embryos at 72 hpf from oral (a, b, c), lateral (a′, b′, c′) and vegetal (a″, b″, c″) views depict the tissues where fluorescence deriving from the fluorescent MO is visible (c–c″). Morphant embryos imaged with microscope (b–b″) present a phenotype similar to uninjected control embryos (a–a″). [file 13227_2018_94_MOESM1_ESM.pdf]

### a Synaptotagmin B positive cells

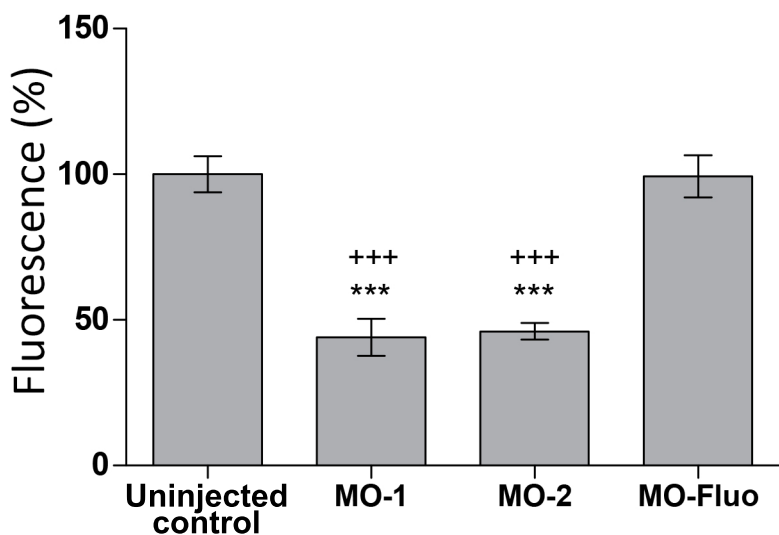

### b Serotonergic neurons

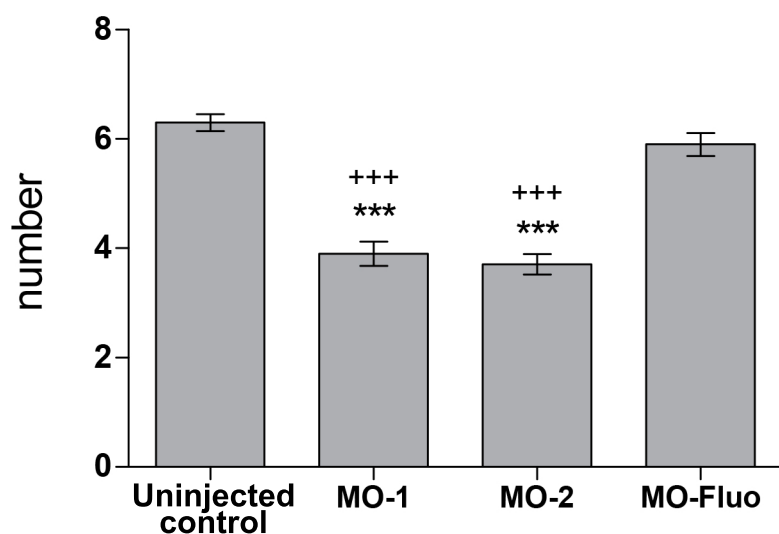

### c Cilia lenght

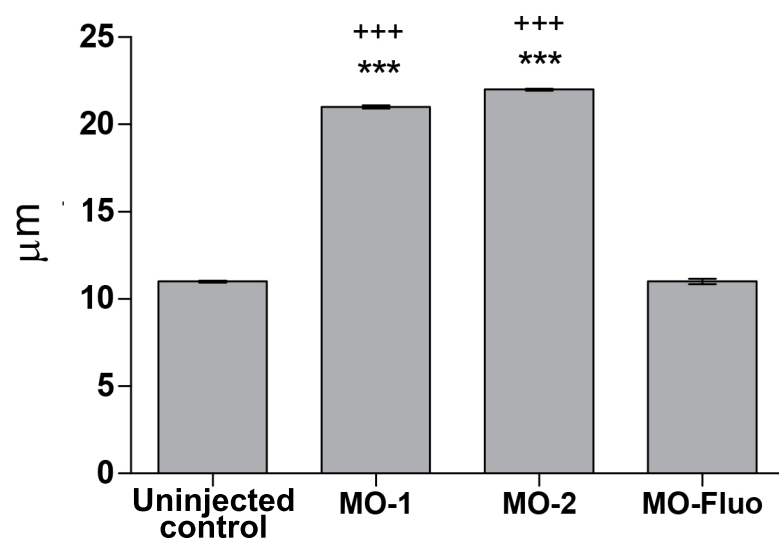

Supplement: Supplementary file 2 — Additional file 2: Figure S2. Statistical analysis of serotonergic neurons number and cilia length in 72 hpf S. purpuratus SoxB2 knock-down experiments. a Embryonic nervous system (Synaptotagmin B by 1e11 immunohistochemistry) of uninjected control, MO-1, MO-2 and MO-Fluo embryos. The fluorescence of 1e11 positive neurons is shown in %, normalized by control uninjected embryos (100%). The intensity of staining from 10 embryos of each group was measured using ImageJ in three independent experiments. b Number of serotonergic neurons observed in six independent experiments using uninjected control, MO-1, MO-2 and MO-Fluo embryos. Serotonergic positive neurons were measured from at least 33 embryos in each experimental group. c Cilia length in uninjected control, MO-1, MO-2 and MO-Fluo embryos measured using the Zeiss confocal laser scanning LSM 510 microscope software. 10–12 cilia from at least 33 embryos were used in three independent experiments. Statistical analysis was performed using Prism 5 GraphPad software: P value versus uninjected controls = *P < 0.05, **P < 0.01, ***P < 0.001, while P value versus MO-Fluo = +P<0.05, ++P < 0.01, +++P < 0.001. [file 13227_2018_94_MOESM2_ESM.pdf]

Control 72 hpf

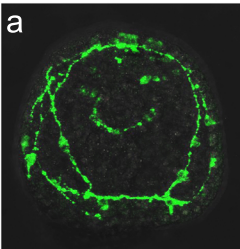

MO-1 72 hpf

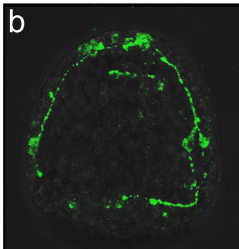

MO-2 72 hpf

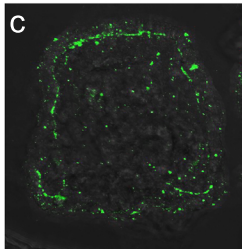

MO-Fluo 72 hpf

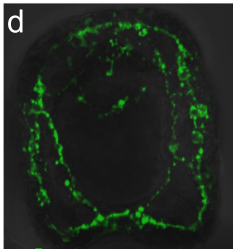

Supplement: Supplementary file 3 — Additional file 3: Figure S4. Injection of MO-Fluo did not affect the development of Synaptotagmin B expressing neurons. [file 13227_2018_94_MOESM3_ESM.pdf]

Control 72 hpf

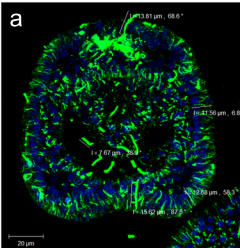

MO-1 72 hpf

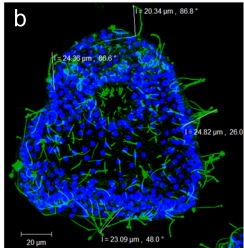

MO-2 72 hpf

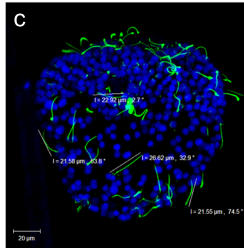

MO-Fluo 72 hpf

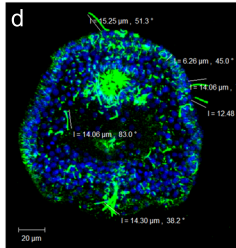

Supplement: Supplementary file 4 — Additional file 4: Figure S3. Analysis of the cilia length in MO-injected embryos performed at 72 hpf pluteus. AcTubulin staining is shown in green and DAPI in blue. a control non injected embryo, b MO-1 injected embryo, c MO-2 injected embryo, d MO-Fluo injected embryo. Measurements of longest cilia length (l) are indicated in white. All cilia length measurements were performed using Ziess LSM Image Browser software. 10–12 cilia from at least 33 embryos in every experimental group were measured; scale bar is 20 µm. [file 13227_2018_94_MOESM4_ESM.pdf]
