# Supplementary material for: SoxB2 in sea urchin development: implications in neurogenesis, ciliogenesis and skeletal patterning
Source: EvoDevo. 2018 Feb 19;9:5. doi: 10.1186/s13227-018-0094-1 (PMC5817722; doi:10.1186/s13227-018-0094-1)
Supplement: Supplementary file 5 — Additional file 5: Table S1. List of oligonucleotides used for qPCR experiments. [file 13227_2018_94_MOESM5_ESM.pdf]

Supplementary Table 1. List of oligonucleotides used for qPCR experiments.

Primer Name		Sequence (5'→3')	Gene ID
Bmp3	Forward	TCCCAGAAAGCGGAGTAAGA	SPU_007822
	Reverse	TCGACAACTTCGTTGCTGAC	
Brn1/2/4	Forward	GTCGCATTAAGCTCGGCTAC	SPU_016443
	Reverse	CAGCGGCTTCAGTTTACACA	
Ef1a	Forward	CTTGGAAAGGGATCGTTCAA	SPU_002050
	Reverse	GCCTGTGAGGTTCCAGTGAT	
Onecut	Forward	TGCAGCTTCTCTGCATACCA	SPU_016449
	Reverse	ACTCCAACATGCCTCCAAAC	
SoxB1	Forward	GGCAACAAGAACAACAGCAA	SPU_022820
	Reverse	AATTGTGCATTTTGGGGTTC	
SoxC	Forward	CATGGTTTGGTCACAAATCG	SPU_002603
	Reverse	TACGGAGATTTCGCCACTTC	
Ubiquitin	Forward	CACAGGCAAGACCATCACAC	SPU_026925
	Reverse	GAGAGAGTGCGACCATCCTC	
Pax2/5/8	Forward	CCAAAGGTGGTGTCGAAGAT	SPU_014539
	Reverse	ATCGAGCTGACACTGGGAAC	
Vegf	Forward	AATGTAGGATCAAGCCACAGG	SPU_014978
	Reverse	GGCATGTTACAGACGCAGTTA	
SM30	Forward	CGTATTGGCTTTGGCCTCTTTC	SPU_000826
	Reverse	GGTAGGTGGTGTGATTGGG	
SM50	Forward	GGAAGAGCTCCAGTCATGAAG	GI:373464
	Reverse	AGGTACCTCACAAACGAAAGC	
